# Health promotion lifestyle interventions for weight management in psychosis: a systematic review and meta-analysis of randomised controlled trials

**DOI:** 10.1186/1471-244X-12-78

**Published:** 2012-07-12

**Authors:** Elena Bonfioli, Loretta Berti, Claudia Goss, Francesca Muraro, Lorenzo Burti

**Affiliations:** 1 Department of Public Health and Community Medicine, University of Verona, Piazzale L.A. Scuro 10, 37134, Verona, Italy

**Keywords:** Body weight, randomised clinical trials, physical health promotion, intervention, meta-analysis, psychosis

## Abstract

**Background:**

Psychiatric patients have more physical health problems and much shorter life expectancies compared to the general population, due primarily to premature cardiovascular disease. A multi-causal model which includes a higher prevalence of risk factors has provided a valid explanation. It takes into consideration not only risks such as gender, age, and family history that are inherently non-modifiable, but also those such as obesity, smoking, diabetes, hypertension, and dyslipidemia that are modifiable through behavioural changes and improved care. Thus, it is crucial to focus on factors that increase cardiovascular risk. Obesity in particular has been associated with both the lifestyle habits and the side effects of antipsychotic medications. The present systematic review and meta-analysis aims at collecting and updating available evidence on the efficacy of non-pharmacological health promotion programmes for psychotic patients in randomised clinical trials.

**Methods:**

We systematically reviewed the randomised controlled trials from 1990 onward, in which psychoeducational and/or cognitive-behavioural interventions aimed at weight loss or prevention of weight gain in patients with psychosis had been compared to treatment as usual. We carried out a meta-analysis and pooled the results of the studies with Body Mass Index as primary outcome.

**Results:**

The results of the meta-analysis show an effect toward the experimental group. At the end of the intervention phase there is a −0.98 kg/m^2^ reduction in the mean Body Mass Index of psychotic subjects. Notably, prevention studies with individual psychoeducational programmes that include diet and/or physical activity seem to have the highest impact.

**Conclusions:**

When compared with treatment as usual in psychotic patients, preventive and individual lifestyle interventions that include diet and physical activity generally prove to be effective in reducing weight. Physical screening and monitoring programmes are well accepted by patients and can be implemented in a variety of settings. A weight loss of 0.98 points in the Body Mass Index corresponds to a loss of 3.12% of the initial weight. This percentage is below the 5% to 10% weight loss deemed sufficient to improve weight-related complications such as hypertension, type II diabetes, and dyslipidemia. However, it is reported that outcomes associated with metabolic risk factors may have greater health implications than weight changes alone. Therefore, in addition to weight reduction, the assessment of metabolic parameters to monitor other independent risk factors should also be integrated into physical health promotion and management in people with mental disorders.

## Background

In comparison to the general population, psychiatric patients, especially those with severe mental illness (SMI) such as schizophrenia or bipolar disorder, have worse physical health and a much shorter life expectancy, due primarily to premature cardiovascular disease (CVD) [1]. This finding has been explained with a multi-causal model including a higher prevalence of risk factors, namely, high blood pressure, high plasma cholesterol, obesity, smoking, diabetes, self-neglect tendencies, unhealthy lifestyles, medication side-effects, and low socio-economic status [2]. Risk factors for cardiovascular morbidity and mortality include those that are inherently non-modifiable (gender, age, family history) and those that are modifiable through behavioural changes and improved care (obesity, smoking, diabetes, hypertension, and dyslipidemia) [3]. Sedentary lifestyles and limited access to low-calorie, high-nutrient foods are but two examples of potential modifiable contributors to obesity in these patients [4].

In order to improve the long-term health of patients with schizophrenia, given their increased risk of CVD, it is imperative to focus attention on factors, such as obesity, which further increase this risk [5]. Among individuals with schizophrenia and affective disorders, obesity is 1.5-2 times higher than in the general population [6]. It has been associated not only with lifestyle habits but also with the side effects of antipsychotic medications, posing serious problems for physical and mental health, including a higher mortality [7]. It has been demonstrated that atypical antipsychotics (in particular, olanzapine and clozapine) contribute to weight gain, albeit with different weight gain liabilities (ziprasidone, for example, has the least) [8,9].

Two systematic reviews and meta-analyses exist on this topic, one [10] on non-pharmacological interventions for antipsychotic-induced weight gain and the other [11] on both pharmacological and non-pharmacological interventions. The first review [10] addresses non-pharmacological interventions for antipsychotic-induced weight gain in patients with schizophrenia-spectrum disorders and it identifies 10 studies [12-21]. According to the authors, adjunctive non-pharmacological interventions are effective in reducing or attenuating antipsychotic-induced weight gain when compared with treatment as usual in patients with schizophrenia-spectrum disorders, and treatment effects may be maintained through follow-ups [10]. The second review [11] focuses on both pharmacological and non-pharmacological interventions for weight reduction in schizophrenic patients or patients with SMI. Evidence from 5 studies on non-pharmacological treatments [12-14,16,19] has been gathered and updated to 2010. In this population, according to the authors, non-pharmacological interventions are possible and show acceptable compliance, although conclusions regarding the intensity of the intervention cannot be made at this stage, and thus there is the need for further randomised controlled trials (RCTs) with long-term follow-ups [11]. However, the role of physical inactivity and poor diet as independent risk factors for CVD infers the need for non-pharmacological, lifestyle interventions regardless of weight loss per se. According to the authors, there is also insufficient evidence to support the general use of pharmacological interventions for weight management in people with schizophrenia [11].

Our systematic review and meta-analysis aims at collecting and updating available evidence on the efficacy of non-pharmacological health promotion programmes for psychotic patients in randomised clinical trials, extending the search period beyond the years covered by the other reviews. The twofold objective is to provide evidence about the physical health of psychiatric patients regarding excessive weight gain, in order to increase awareness of this issue in both the scientific community and the relevant stakeholders, and to expand the setting up of effective intervention programmes. The main outcome variable taken into consideration is Body Mass Index (BMI), as specified and explained in Methods.

## Methods

We reviewed RCTs from 1990 onward, which pertain to psychoeducational and/or cognitive-behavioural interventions aimed at weight loss or prevention of weight gain in patients with psychosis. The present work is part of a larger systematic review (to be published) which, after a wide-scale search of the literature on physical health promotion interventions in psychiatric patients, considers not only weight management interventions but also interventions targeted at smoking, diet, physical activity, and HIV prevention.

### Eligibility criteria

We considered as eligible those randomised clinical trials on the efficacy of weight management interventions which had been published in English from 1990 up to the date of the search. They included at least 50% of adult subjects between the ages of 18 and 65 who, according to the International Classification of Disorders (ICD) [22] (codes F20-25, F28-31, F32.3, F33.3), had been diagnosed with: schizophrenia and related disorders (schyzotypal disorder, delusional disorder, acute and transient psychotic disorders, induced psychotic disorder, schizoaffective disorder, other nonorganic psychotic disorders, unspecified nonorganic psychosis), bipolar affective disorder, manic episode, depressive episode with psychotic symptoms or depressive disorder with psychotic symptoms. We included in our review those studies based on programmes aimed at weight reduction or prevention of weight gain through cognitive-behavioural, psychoeducational, nutritional or physical activity-based interventions. We excluded pharmacological interventions used to reduce weight.

The primary outcome considered is mean BMI (kg/m^2^) of the groups at endpoint or change in BMI. Although the use of BMI has limitations, because it does not distinguish between fat and lean mass nor does it adjust for age or sex, it is generally accepted as a reasonable guide for clinical and epidemiologic purposes [23].

### Search strategy

Only relevant studies from 1990 onward were considered, since previously published reviews had already covered the years prior to that date [10,11]. The following search terms were used: *psychosis* and *intervention* and *health promotion* or *health education* or *physical health* or *smoking* or *weight gain* or *exercise* or *HIV risk* or *AIDS* or *infection*. The searches were made on: a) Pubmed, Web of Science, PsycInfo, Embase databases, b) the Cochrane Library, c) reference section of retrieved papers and review articles. The last search, performed in May 2010, was later updated to cover the period up to and including December 2011. To satisfy the specific aims of this review, only studies relevant to weight gain were selected from the results.

### Study selection

Records retrieved from the search were screened by title. Possible inclusions were screened by abstract. Full text and relevant papers were then selected. Conference abstracts and letters to the editor were excluded. For the meta-analysis, we considered eligible RCTs on weight management interventions. It was decided to contact the authors of the studies that did not include sufficient data for statistical analysis. Two reviewers discussed dubious cases in order to reach an agreement on the inclusion of studies.

### Data extraction

Relevant data for the systematic review and for the meta-analysis were extracted by 2 of our authors using the format: 1) inclusion criteria; 2) characteristics of the sample (diagnosis of the subjects, duration of the illness, i.e., first episode or chronic psychosis); 3) number of subjects that entered the analysis in the experimental and in the control group; 4) number of drop-outs; 5) type of intervention (prevention of weight gain or weight loss, group or individual, cognitive-behavioural or psychoeducational, diet and/or physical activity); 6) mean and standard deviation (SD) of the outcome considered (BMI or BMI change) in both the experimental and the control group at baseline and endpoint. We assessed the risk of bias based on the recommendations of the Cochrane Handbook for Systematic Reviews of Interventions [24].

The summary measure considered is the mean difference (MD) of the primary outcome (BMI or BMI change) in the experimental and the control group at baseline and endpoint. The statistical method used for the meta-analysis is inverse variance, with a random-effects approach, utilising RevMan (Cochrane Collaboration’s software for meta-analyses). Heterogeneity is investigated by the I^2^ statistic [25].

We hypothesized that the methodological quality of the studies and some characteristics of the samples and procedures used might influence the variability of results. As a means of investigating the heterogeneity of results and answering specific questions about particular patient groups and types of interventions, we decided to carry out subgroup analyses, making the following comparisons:

first episode psychosis vs. chronic psychosis;

weight gain prevention vs. weight loss;

group intervention vs. individual intervention;

use of cognitive-behavioural therapy (CBT) vs. psychoeducation;

physical activity vs. no physical activity;

diet vs. no diet.

We also decided to analyse the differences between experimental and control groups regarding the number of drop-outs. We determined to assess the robustness of the findings through sensitivity analyses.

## Results

**Figure 1:**
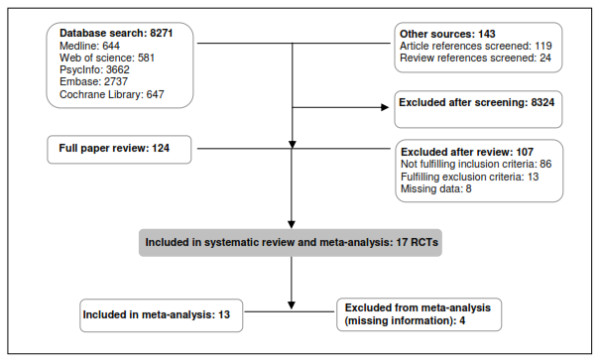
Stages of systematic review and meta-analysis.

Seventeen RCTs were retrieved from the search. Five did not provide sufficient information for meta-analysis [18,26-29]. However, only 4 studies were excluded [18,27-29] because in one case [26] the authors supplied the missing data. For one study we had to impute SDs from the pool of data [30]. Therefore, 13 RCTs were eligible for inclusion [12-17,19-21,26,30-32]. More details on the screening process can be found in Figure 1. 

Five studies included CBT interventions whereas 8 included psychoeducational or other types of intervention. Four studies evaluated an individual intervention and 9 evaluated a group intervention. Four studies included some physical activity and 3 included a diet. In all the studies except one [31], the control group was given treatment as usual and in some cases, brief nutritional information [14-17,20].

Interventions lasted between 2 and 12 months. The mean duration of interventions (excluding the one of Brown and Smith [26] who did not provide information) is about 18 weeks. Three studies reported follow-up periods of 2 to 3 months [12,14,20]. The trials were carried out in Europe, United States of America (USA), Asia and Australia.

More detailed information on the characteristics of the studies is reported in Table 1 and in the paragraph “Heterogeneity of studies”.

**Table 1:** Characteristics of randomised controlled trials of lifestyle interventions for weight gain in psychosis

**Study (year)**	**Sample size**	**Participants/ setting**	**Diagnosis**	**Antipsychotic medication**	**Primary outcomes**	**Intervention**	**Control**	Duration*	Followup**
Álvarez Jiménez et al. (2006) [15]	(a) 28	Outpatients	Schizophrenia, schizophreniform disorder, schizoaffective disorder, delusional disorder, brief reactive psychosis, or psychosis not otherwise specified (NOS)	Olanzapine, risperidone, haloperidol	- Body weight	10 to 14 individual sessions (weight check, agenda setting, review of self monitoring records, homework assignments) provided by clinical psychologists	Usual care + nonstructured information about weight gain and encouragement to limit food intake and/or increase physical activity	12	0
	(b) 33				- BMI change				
					- Percentage of patients whose weight increased by more than 7 % of the initial weight				
Brar et al. (2005) [13]	(a) 35	Outpatients or stable long-term inpatients	Schizophrenia (38), Schizoaffective disorder (33)	Risperidone	Body weight change	20 group-based behavioural treatment sessions for weight loss (manual driven didactic programme)	Usual care	14	0
	(b) 37			Concomitant medications: sedative-hypnotics, antidepressants					
Brown & Smith (2009) [29]	(a) 15	Outpatients	Schizophrenia (11), bipolar disorder (5), depression (9), borderline personality disorder (3)	Weight gain drugs (not specified)	Body weight change	5 semistructured health promotion sessions using an operational manual based on motivational interviewing, education, diary keeping, and facilitation of access to mainstream facilities, facilitated by mental health key workers	Usual care	N/A	0
	(b) 11								
Evans et al. (2005) [14]	(a) 29	Outpatients	Schizophrenia (16), Schizoaffective disorder (11), schizophreniform psychosis (10), bipolar disorder (8), depression (5)	Olanzapine	- Body weight	6 individual nutritional education sessions conducted by an accredited practicing dietician	Passive nutritional education from the booklet “Food for the mind”	12	12
	(b) 22				- BMI change				
					- Waist circumference change				
Forsberg et al. (2008) [27]	(a) 27	Supported housing facilities	Schizophrenia (23), bipolar disorder (3), other psychotic disorders (7), other psychiatric diagnoses (8)	Antipsychotic medication	- Weight	Programme for healthy living: 2 sessions weekly focusing on the cooking of good nourishing food and on physical activity (indoor and outdoor activities) lead by a circle leader (no training in mental health field and no own experience of working with person with psychiatric disabilities but has a personal interest in healthy food and experience as a fitness instructor)	“aesthetic study circle” (learn and practice artistic techniques)	52	0
	(b) 19				- Waist				
					- BMI				
					- Physiological values				
Khazaal et al. (2007) [20]	(a) 31	Outpatients	Schizophrenia and schizoaffective disorders (73.8 %), bipolar disorder (8.2 %), schizotypal disorder (6.6 %), other (11.5 %)	Olanzapine, risperidone, clozapine, quetiapine, amisulpride, classical antipsychotics	- Body weight	12 2-hour group sessions weekly (motivational interview), tasting sessions, psychoeducation on links between weight gain and antipsychotics, food intake moderation prescribed, provided by two psychologists	Brief Nutritional Education (one informative 2 hour group session)	12	3
	(b) 30				- BMI				
					- Eating and weight-related cognitions (MAC-R) - Binge eating simptomatology (SCID-IV)				
Kwon et al. (2006) [16]	(a) 33	Outpatients	Schizophrenia or schizoaffective disorder	Olanzapine	- Body weight	Diet and exercise management programme based on cognitive and behavioural therapy, nutritional education, diary and exercise lead respectively by a dietician and an exercise coordinator	Usual care + recommendations as to physical activity and eating	12	0
	(b) 15				- BMI				
Littrell et al. (2003)	(a) 35	Outpatients	Schizophrenia (54), schizoaffective disorder (16)	Olanzapine	- Body weight	16 1-hour psychoeducation classes using the "Solutions of wellness" modules ("Nutrition, wellness and living a healthy lifestyle", "Fitness and exercise") held by a clinician	Usual care + olanzapine	16	8
	(b) 35			Concomitant medications: lithium, valproate, SSRI	- BMI				
Mauri et al. (2008) [28]	(a) 21	Outpatients	Bipolar I disorder (41), bipolar II disorder (2), depressive disorder with psychotic symptoms (1)	Olanzapine	- Body weight	dietary group programme for weight control: 30-minutes psychoeducational meetings + diet	N/A	12	0
	(b) 27				- BMI				
McKibbin et al. (2006) [17]	(a) 32	Board-and-care and community clubhouse	Schizophrenia (48), schizoaffective disorder (9)	Antipsychotics	- Body weight	24 weekly, 90 min sessions addressing diabetes education, nutrition, and lifestyle exercise conducted by healthcare providers, dieticians, and diabetes educators	Usual care + 3 brochures from American Diabetes Association	24	0
	(b) 32				- BMI				
					- Waist circumference change				
Milano et al. (2007) [26]	(a) 22	Outpatients	Schizophrenia or manic episodes in bipolar disease	Olanzapine	- Body weight change	Psychoeducational programme with information on correct alimentary practices and personal health; diet (reduction of 500 kcal/ die); programme on physical exercise (3/wk, 30-60 min)	Regular diet, no physical activity	8	0
	(b) 14								
					- BMI				
Weber & Wyne (2006) [19]	(a) 8	Outpatients	Schizophrenia or schizoaffective disorder	One oral atypical antipsychotic	- Body weight	1-hour group session based on cognitive- behavioural strategies to promote risk reduction (with food and activity diary) provided by a trained psychiatric nurse practitioner supervised weekly	Usual care	16	16
	(b) 9				- BMI				
					- Waist-hip ratio - Blood glucose level				
Wu et al. (2007) [21]	(a) 28	Hospitalized patients	Schizophrenia	Clozapine	- Body weight	Dietary control by a registered dietician. 1-hour physical activity sessions 3 times a week	N/A	24	0
	(b) 28				- BMI				
					- Body fat				
					- Waist-hip ratio				

(a) experimental group.

(b) control group.

*number of weeks.

**follow-up assessment, number of weeks after the end of intervention.

### Risk of bias

**Figure 2:**
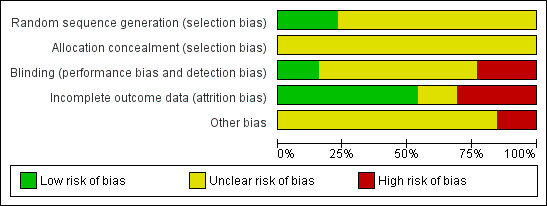
Risk of bias graph.

The assessment of risk of bias is presented in Figure 2 and Additional file 1: Table S1. Only few studies describe random sequence generation [15,26,31] while none describes allocation concealment. None of the trials has a double-blind design. Three of them are single-blind trials [15,19,26], however in one of the studies it is stated that blinding of assessors was occasionally difficult to achieve [15]. Mauri et al. [32] and Khazaal et al. [20] have an open-label design. The remaining studies do not report information on blinding. Four studies report only data for completers [14,17,19,21] and thus do not address incomplete data adequately. Seven studies included all the subjects in the analyses or provided intention-to-treat analysis [12,13,15,16,26,30,32]. It should be noted that poor quality randomisation can produce unequal groups at baseline: Such might be the case of some studies in this review [20,26]. In Weber & Wyne [19] subjects were given $5 for each complete visit. 

### Results for all interventions

**Figure 3:**
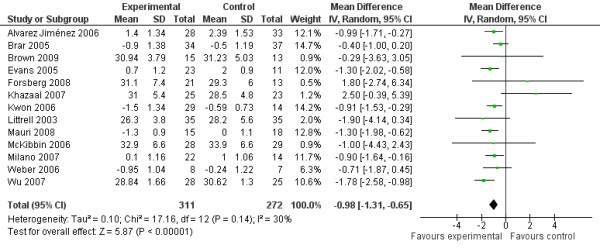
Efficacy of lifestyle interventions (Experimental) vs. treatment as usual (Control) for weight management in psychosis.

The main analysis (Figure 3) shows an effect toward the experimental groups, with a reduction in mean BMI of −0.98 kg/m^2^, compared to the control groups (95% CI: -1.31 kg/m^2^ to −0.65 kg/m^2^). A statistical test for heterogeneity failed to suggest substantial heterogeneity.

### Drop-outs

There are no significant differences in the number of drop-outs between experimental and control groups (OR = 1.08 95% CI 0.67 to 1.73), except for Evans et al. [14], which has a greater number of drop-outs in the control group. In that trial, 6 of the 11 drop-outs in the control group are due to discontinuation or non-adherence regarding olanzapine treatment (introduced at the beginning of treatment) and 5 are due to non-contactable issues.

### Subgroup analyses

Subgroup analyses (Table 2) demonstrate that studies about weight gain prevention with individual psychoeducational programmes that include diet and/or physical activity seem to have the highest impact. However, it is also true that a greater sample size relates to a greater heterogeneity and smaller effect size, except for the comparison between CBT and psychoeducation, where the contrary is true. Concerning the length of illness, both studies with chronic patients and first-episode patients seem to have similar effects. The study with mixed sample seems to be more successful in the reduction of weight.

**Table 2:** Subgroup analyses

**Subgroups**	**Number of studies**	**Number of subjects**	MD (CI)*	**I**^**2**^
Weight gain prevention	4	(a) 108 (b) 93	- 1.09 (−1.51, -0.68)	0 %
Weight loss	9	(a) 203 (b) 179	−0.86 (−1.38, -0.33)	49 %
Group intervention	9	(a) 203 (b) 189	−0.70 (−1.24, -0.15)	37 %
Individual intervention	4	(a) 108 (b) 83	−1.20 (−1.57, -0.83)	8 %
CBT	5	(a) 124 (b) 114	−0.66 (−1.15, -0.16)	41 %
Psychoeducation	8	(a) 187 (b) 158	−1.28 (−1.64, -0.93)	0 %
First-episode psychosis	1	(a) 28 (b) 33	−0.99 (−1.71, -0.27)	N/A
Chronic psychosis	11	(a) 260 (b) 228	−0.92 (−1.34, -0.49)	39 %
Mixed sample	1	(a) 23 (b) 11	−1.30 (−2.02, -0.58)	N/A
Physical activity	4	(a) 93 (b) 90	−1.22 (−1.59, -0.85)	2 %
No physical activity	9	(a) 218 (b) 182	−0.75 (−1.22, -0.28)	27 %
Diet	3	(a) 65 (b) 57	−1.31 (−1.78, -0.83)	21 %
No diet	10	(a) 246 (b) 215	−0.80 (−1.19, -0.42)	20 %

* Random-effects method, 95% CI.

(a) experimental group.

(b) control group.

### Sensitivity analyses

We performed sensitivity analyses to assess the robustness of the findings with respect to: a) the choice of fixed-effect or random-effects methods, b) the exclusion of studies that provided only BMI values instead of BMI changes, c) the exclusion of trials with a duration of less than 12 weeks or more than 1 year, d) the exclusion of trials with control groups that are not completely inactive.

### Heterogeneity of studies

Even though our analysis did not detect statistically significant heterogeneity, study samples are heterogeneous according to mean initial weight, length of illness, diagnosis, and pharmacological therapy. In 5 of the 13 studies, both the experimental and the control group at baseline have a mean BMI in the obesity category (30–39.9 kg/m^2^) [17,19,21,26,32]. In 3 studies the mean BMI of the experimental and control subjects falls into the overweight category (25–29.9 kg/m^2^) [12,14,16]. One study included subjects with a mean BMI in the normal category [15]. In Milano et al. [30], Forsberg et al. [31] and Khazaal et al. [20], the control group falls into a lower category of BMI when compared to the experimental group. In the remaining study mean BMI at baseline is not known [13], but one of the inclusion criteria is BMI > 26. Such differences in weight at the beginning of intervention may have a role in influencing the total intervention effect. It is important to note that most studies on weight gain prevention included normal weight or overweight subjects whereas studies on weight loss included obese or overweight subjects. We included only those studies with at least 50% psychotic patients, although most authors include other diagnoses in their studies. However, all patients take antipsychotics. In one of the studies the sample consisted of drug-naïve first-episode patients [17]. The length of illness varies (from first-episode patients to chronic patients) with a maximum length of about 26 years. The mean age is also variable. At times in the same study, some patients take typical antipsychotics while others take atypical antipsychotics that may be for specific study purposes only [12,14,15,30]. Some authors do not specify the type of antipsychotic therapy used in their study [17,19,27,29].

Trials are also heterogeneous according to objectives (weight gain prevention or weight loss), type and duration of interventions (presence or absence of diet and/or physical activity, psychoeducation or CBT, duration, follow-up etc.), background/training of professionals, control group (treatment as usual, informative booklets or sessions, treatment not specified), and methodological sophistication. It must be stated that it is very difficult to properly perform RCTs in this field due to the amount of external confounding influences [33]. A number of studies lack a description of the randomisation procedure. For further information on randomisation and methodological characteristics of the studies refer to Additional file 1: Table S1.

## Discussion

The results of the meta-analysis show that when compared to treatment as usual, preventive and individual lifestyle interventions including diet and physical activity generally reduce weight in psychotic patients by −0.98 BMI points. Such weight loss corresponds to a loss of 3.12% of the initial weight (31.36 kg/m^2^, except [13], for which we do not have baseline BMI data). However, a weight loss of 5% to 10% is the criterion for success proposed by the National Institute of Health/National Heart Lung and Blood Institute, the World Health Organization, and the Dietary Guidelines for Americans. Nonetheless, even though a 3.12% loss is below percentages reported as sufficient to improve weight-related complications including hypertension, type II diabetes, and dyslipidemia [34-36], Gabriele et al. [37] state that in individuals taking atypical antipsychotics, outcomes associated with metabolic risk factors may have greater health implications than weight changes alone. Therefore, in addition to weight measurements, the assessment of metabolic parameters should also constitute integral part of physical health monitoring in people with mental disorders, because it can provide meaningful information on weight-related health status.

Poor results of weight management interventions can be due to their short duration or to their lack of follow-up sessions. The mean duration of interventions in the trials included is only about 18 weeks with a median of 12 weeks. The lack of follow-up is evidenced by the fact that out of the 13 trials, only 3 provided follow-up sessions, one after 2 and the other two after 3 months [12,14,20]. Álvarez Jiménez et al. [15] and McKibbin et al. [17] did expand their studies with a 2-year and a 6-month follow-up respectively [38,39]. It is significant that in the general population, obese patients treated with behaviour therapy for 20 to 30 weeks typically regain about 30% to 35% of their lost weight in the year following treatment [40]. Although studies do not yet provide clear evidence as to the optimum length of engagement in these programmes, experience in the general population suggests that lifestyle change needs to be permanent [41]. Thus, intervention programmes should last at least 20 weeks and should provide follow-ups consisting of booster sessions for behavioural control and for diet and physical activity control. In any case, attention should be brought to the fact that an 18 week follow-up is a very short period of time compared to the estimated 25 years of life which are lost in these patients due to metabolic syndrome [42].

### Strengths of the study

Scientific research in this field is relatively new. Differences between countries regarding resources, physical and mental health services, and attitudes toward physical and mental illness can be expected to produce wide variations in physical health care for patients with SMI [43]. This review contributes to the update of available evidence and it highlights the fact that in recent years, interest of both researchers and clinicians toward the physical health of mental patients has increased. Thus, we are witnessing a rise in the number of studies on physical comorbidity in this population. Most of the studies are conducted in outpatient settings located in Europe or USA, except for the one inpatient setting located in Asia. More and more mental patients are treated in the community, where they may receive less physical health attention than hospitalised patients, and precisely for this reason it is important to focus on the health status of outpatients. It is also important to point out that the homogeneity in the setting of the studies is useful for testing feasibility of interventions in routine contexts. Health promotion interventions seem to meet the current requirements of psychiatric services, which prove to be the right setting for physical health care in this particular population. According to Chaudhry and colleagues [44], the physical health of hospitalised patients is closely monitored (although some simple and relevant indexes, e.g., waist circumference, are usually not recorded routinely). However, in the case of adverse effects, switching antipsychotic therapy is more likely to be considered for neurological side effects rather than for abnormal lab findings such as those of the metabolic syndrome. Dangers arising from metabolic disturbances are not yet fully appreciated. Patients in the community are an urgent priority for improved monitoring and long-term management [44]. This seems feasible. In fact, we found a lack of statistically significant differences in the number of drop-outs between experimental and control groups, which suggests that dropping-out depends on factors not related to the feasibility and acceptability of the interventions per se. Physical screening and monitoring programmes may be well accepted by patients and can be implemented in a variety of settings.

### Limitations

#### Limitations at study level

A notable limitation at study level regards heterogeneity, which has been presented in Results (“Heterogeneity of studies”).

#### Limitations at review level

We conducted a methodical search of literature to find every relevant trial for our research question. However, we do not exclude the possibility of reporting bias.

It must also be taken into account that the following factors may have influenced the results of the meta-analysis:

– few studies describe the randomisation method;

– no study describes allocation concealment;

– few studies describe blinding;

– some studies only report data for completers or do not report the reasons for missing data;

– in one study subjects received money for their participation ( Additional file 1: Table S1).

With respect to informativeness, 4 studies out of 17 did not include data essential to carry out a meta-analysis, therefore we were forced to exclude them from our review. Other papers lack information on reasons for missing data and do not use approaches to deal with missing outcome data, such as intention-to-treat analysis or “last observation carried forward”. This may be a problem because missing outcome data, due to attrition (drop-out) during the study or exclusions from the analysis, raise the possibility of a bias in the observed effect estimate [24].

### Implications for future research

As to research methodology, more comparable and more rigorous research methods would be advantageous. This would facilitate the comparisons and the testing of possible confounding factors and methodological biases on the outcome of interventions, and subsequently on the result of meta-analyses. Greater sample sizes and more studies would be necessary. More information on the cost-effectiveness of interventions would also be of use. Information on missing data in trials would need to be provided as it is crucial for the understanding of their influence on outcome, efficacy, and effectiveness of proposed interventions.

As to intervention methodology, it is important for professionals to have a more homogeneous background or at least a psychiatric professional background. Besides weight measurement, the measurement of both obesity-related physical health parameters (such as waist circumference) and metabolic parameters, should be considered mandatory in future protocols. The individual components of the metabolic syndrome, as well as some other non-metabolic parameters, should be checked at baseline and measured regularly thereafter (especially in drug- naïve first-episode patients, children and adolescents) [45]. In the present review some, but not all trials included such secondary outcomes.

## Conclusions

The studies reviewed are still to be considered preliminary. Our hope is that this paper will be a small but inspiring step toward the development of larger and much longer studies. Although the findings of this paper are to be regarded as of a preliminary nature, it is hoped that they will be instrumental in bringing this problem to the attention of medical practitioners world-wide. It is clear that more research is required, especially with an increase in the number of parameters under study. However, it is also clear that patients with SMI are willing to participate and remain in health promotion programmes. Consequently, these programmes may have positive effects in diminishing risk factors, with relevant impact on clinical practice.

## Abbreviations

BMI: Body mass index; BT: behavioural therapy; CBT: Cognitive-behavioural therapy; CI: Confidence interval; CVD: Cardiovascular disease; ICD: International classification of diseases; MD: Mean difference; N/A: Not available; NOS: Not otherwise specified; OR: Odds ratio; RCT: Randomised controlled trial; SMI: Severe mental illness; SD: Standard deviation; UC: usual care; USA: United States of America.

## Competing interests

The authors declare that they have no competing interests.

## Authors’ contributions

CG carried out the review. FM updated it. CG, LBe, FM, and EB implemented data extraction. EB performed the meta-analysis and drafted the manuscript. LBu conceived the study, participated in its design and coordination, and participated in the writing of the manuscript. All authors read and approved the final manuscript.

## Pre-publication history

The pre-publication history for this paper can be accessed here:

http://www.biomedcentral.com/1471-244X/12/78/prepub

## Supplementary Material

Additional file 1 Table S1.Risk of bias table. Click here for file
