# Supplementary material for: Health promotion lifestyle interventions for weight management in psychosis: a systematic review and meta-analysis of randomised controlled trials
Source: BMC Psychiatry. 2012 Jul 12;12:78. doi: 10.1186/1471-244X-12-78 (PMC3549787; doi:10.1186/1471-244X-12-78)
Supplement: Additional file 1 — Table S1.Risk of bias table. [file 1471-244X-12-78-S1.doc]

Table S1 **Risk of bias table**

| **Item** | **Judgement** | **Description** |
| --- | --- | --- |
| **Alvarez Jiménez et al. 2006** [15] | | |
| **Adequate sequence generation?** | Yes | Patients were randomly assigned by computer-generated blocks of 4 random numbers. The randomisation was performed by a member of the team not involved with either the assessments or the treatments. |
| **Allocation concealment?** | Unclear | No description of allocation concealment. |
| **Blinding?** | No | The study was designed as a single-blind randomised controlled trial, with research assessors and patients intended to be blind to the intervention status. The staff members performing the assessments were not involved in implementing any aspect of the intervention. However, the single blind for assessors was occasionally difficult to achieve because both intervention groups were treated by different clinicians, a feature that was difficult to conceal from assessors. |
| **Incomplete outcome data addressed?** | Yes | No drop-outs. All subjects were included in data analysis. |
| **Free of other bias?** | Unclear | Unclear. |
| **Brar et al. 2005** [13] | | |
| **Adequate sequence generation?** | Unclear | No description of sequence generation. |
| **Allocation concealment?** | Unclear | No description of allocation concealment. |
| **Blinding?** | Unclear | No description of blinding. |
| **Incomplete outcome data addressed?** | Yes | Seventy-two patients were randomised. One patient in the behavioural therapy (BT) group withdrew consent, did not receive study medication, and was removed from all analyses. 71 patients were included in the ITT analysis. 12 patients in the BT group and 9 in the usual care(UC) group dropped out (n = 21). Most common reasons for discontinuation were adverse events (BT = 1, UC = 1), insufficient response (BT = 1, UC = 0), and lost to follow-up (BT = 2, UC = 1). No reasons given for the remaining 9 drop-outs. |
| **Free of other bias?** | Unclear | Unclear. |
| **Brown & Smith 2009** [29] | | |
| **Adequate sequence generation?** | Yes | Subjects were randomised using a hidden computer-generated random number programme. |
| **Allocation concealment?** | Unclear | No description of allocation concealment. |
| **Blinding?** | Yes | Pre and post intervention measurements were made by the same rater who was blind to the interviewees’ status in the study. |
| **Incomplete outcome data addressed?** | Yes | Drop-outs in the experimental group: 5/15. Various personal reasons, e.g., too busy, not useful. Drop-outs in the control group: 0/11. ITT analysis. |
| **Free of other bias?** | No | The experimental and control group BMI are significantly different at baseline (ss of the control group weight more). |
| **Evans et al. 2005** [14] | | |
| **Adequate sequence generation?** | Unclear | No description of sequence generation. |
| **Allocation concealment?** | Unclear | No description of allocation concealment. |
| **Blinding?** | Unclear | No description of blinding. |
| **Incomplete outcome data addressed?** | No | Seventeen participants were withdrawn from the study. 11/22 of these withdrawals (65 %) were from the control group and were withdrawn due to ceasing or non-compliance with olanzapine (n = 6), or due to being non-contactable (n = 5). The 6/29 withdrawals from the intervention group were due to ceasing olanzapine (n = 1), being non-contactable (n = 1), pregnancy (n = 1), moving from the area (n = 1), and not wanting to participate (n = 2). |
| **Free of other bias?** | Unclear | Unclear. |
| **Forsberg et al. 2008** [27] | | |
| **Adequate sequence generation?** | Yes | The randomization was conducted at group level by the drawing of lots by a person not involved in the project. |
| **Allocation concealment?** | Unclear | No description of allocation concealment. |
| **Blinding?** | Unclear | No description of blinding. |
| **Incomplete outcome data addressed?** | Unclear | 3 drop-outs in the experimental group and 2 in the control group on a total of 46 subjects randomised. Informed reasons for cessation during the study were: dissatisfaction of their study circle, health problem, job, and no reason mentioned. Authors state they have performed an-intention-to-treat analysis but the article reports only incomplete data. |
| **Free of other bias?** | Unclear | Some of the control groups increased their physical activities on an individual and group level, e.g., taking part in indoor bandy tournaments and eating somewhat healthier food. This might be a positive development for their health, but can be seen as a drawback for the study. |
| **Khazaal et al. 2007** [20] | | |
| **Adequate sequence generation?** | Unclear | No description of sequence generation. |
| **Allocation concealment?** | Unclear | No description of allocation concealment. |
| **Blinding?** | No | Open-label design. |
| **Incomplete outcome data addressed?** | Unclear | 6/31 drop-outs in the experimental group and 2/30 in the control group, reasons unclear. Missing data were excluded from the analysis. |
| **Free of other bias?** | No | The experimental and control group BMI are significantly different at baseline (BMI is lower in the control group). |
| **Kwon et al. 2006** [16] | | |
| **Adequate sequence generation?** | Unclear | No description of sequence generation. |
| **Allocation concealment?** | Unclear | No description of allocation concealment. |
| **Blinding?** | Unclear | No description of blinding. |
| **Incomplete outcome data addressed?** | Yes | Of the 48 patients enrolled, the data on 5 subjects were incomplete with regard to body weight (4/33 subjects in the experimental group and 1/15 in the control group). Missing data were imputed with “last observation carried forward”. |
| **Free of other bias?** | Unclear | Unclear. |
| **Littrell et al. 2003** [12] | | |
| **Adequate sequence generation?** | Unclear | No description of sequence generation. |
| **Allocation concealment?** | Unclear | No description of allocation concealment. |
| **Blinding?** | Unclear | No description of blinding. |
| **Incomplete outcome data addressed?** | Yes | No drop-outs. All subjects were included in data analysis. |
| **Free of other bias?** | Unclear | Unclear. |
| **Mauri et al. 2008** [28] | | |
| **Adequate sequence generation?** | Unclear | No description of sequence generation. |
| **Allocation concealment?** | Unclear | No description of allocation concealment. |
| **Blinding?** | No | Open-label design. |
| **Incomplete outcome data addressed?** | Yes | A total of 16/48 patients dropped out before study end: the reasons for discontinuations were patient decision in 12 cases (1 before randomization, 3 in the experimental group, 8 in the control group), physician decision in 3 (all in the experimental group), and ineligibility due to exclusion criteria in 1 (in the control group). No specific reasons for drop-out are given. ITT analysis. |
| **Free of other bias?** | Unclear | Unclear. |
| **McKibbin et al. 2006** [17] | | |
| **Adequate sequence generation?** | Unclear | No description of sequence generation. |
| **Allocation concealment?** | Unclear | No description of allocation concealment. |
| **Blinding?** | Unclear | No description of blinding. |
| **Incomplete outcome data addressed?** | No | A total of 7 randomly assigned participants (4/32 in the experimental group; 3/32 in the control group) did not complete the study for the following reasons: inpatient hospitalization (n = 2), unable to complete the follow-up assessment (n = 1), relocation (n = 1), death prior to intervention commencement (n = 1), psychiatric decompensation (n = 1), and lack of interest (n = 1). Only data for completers are reported. |
| **Free of other bias?** | Unclear | Unclear. |
| **Milano et al. 2007** [26] | | |
| **Adequate sequence generation?** | Unclear | No description of sequence generation. |
| **Allocation concealment?** | Unclear | No description of allocation concealment. |
| **Blinding?** | Unclear | No description of blinding. |
| **Incomplete outcome data addressed?** | Yes | No drop-outs. All subjects were included in data analysis. |
| **Free of other bias?** | Unclear | It is unclear whether there are significant differences at baseline between the experimental and control group or not. |
| **Weber & Wyne 2006** [19] | | |
| **Adequate sequence generation?** | Unclear | No description of sequence generation. |
| **Allocation concealment?** | Unclear | No description of allocation concealment. |
| **Blinding?** | Yes | Subjects were weighed and measured by the blind researcher. |
| **Incomplete outcome data addressed?** | No | Two drop-outs in the control group beacuse of worsening psychosis. Only data for completers is reported. |
| **Free of other bias?** | Unclear | Subjects were given 5$ for each completed visit. |
| **Wu et al. 2007** [21] | | |
| **Adequate sequence generation?** | Unclear | No description of sequence generation. |
| **Allocation concealment?** | Unclear | No description of allocation concealment. |
| **Blinding?** | Unclear | No description of blinding. |
| **Incomplete outcome data addressed?** | No | Three drop-outs from the control group because they were discharged from the hospital. Only data for completers are reported. |
| **Free of other bias?** | Unclear | Unclear. |
